# Formation of Cyclobutane Pyrimidine Dimers after UVA Exposure (Dark-CPDs) Is Inhibited by an Hydrophilic Extract of *Polypodium leucotomos*

**DOI:** 10.3390/antiox10121961

**Published:** 2021-12-07

**Authors:** Mikel Portillo-Esnaola, Azahara Rodríguez-Luna, Jimena Nicolás-Morala, María Gallego-Rentero, María Villalba, Ángeles Juarranz, Salvador González

**Affiliations:** 1 Department of Biology, Faculty of Sciences, Instituto Ramón y Cajal de Investigación Sanitaria (IRYCIS), Autónoma University of Madrid (UAM), 28049 Madrid, Spain; mikel.portillo@uam.es (M.P.-E.); jimena.nicolas@uam.es (J.N.-M.); maria.gallego@estudiante.uam.es (M.G.-R.); 2 Cantabria Labs, Innovation and Development, 28043 Madrid, Spain; azahara.rodriguez@cantabrialabs.es; 3 Cantabria Labs, Medical Affairs Department, 28043 Madrid, Spain; maria.villalba@cantabrialabs.es; 4 Department of Medicine and Medical Specialties, Alcalá de Henares University, 28805 Madrid, Spain

**Keywords:** cyclobutane pyrimidine dimers, dark cyclobutene pyrimidine dimers, ultraviolet radiation, *Polypodium leucotomos*, photoprotection, melanocytes, skin cancer

## Abstract

Exposure to sun and especially to ultraviolet radiation (UVR) exerts well known detrimental effects on skin which are implicated in malignancy. UVR induces production of cyclobutane pyrimidine dimers (CPDs), immediately during exposure and even hours after the exposure, these latter being called dark-CPDs, as consequence of the effects of different reactive species that are formed. Fernblock^®^ (FB), an aqueous extract of *Polypodium leucotomos*, has proven to have photoprotective and antioxidant effects on skin. The aim of our work was to investigate the potential photoprotective effect of FB against dark-CPD formation. Murine melanocytes (B16-F10) were exposed to UVA radiation and the production of dark-CPDs and different reactive oxygen and nitrogen species (ROS and RNS) was measured. Significant dark-CPD formation could be seen at 3 h after UVA irradiation, which was inhibited by the pre-treatment of cells with FB. Formation of nitric oxide, superoxide and peroxynitrite was increased after irradiation, consistent with the increased CPD formation. FB successfully reduced the production of these reactive species. Hence, these results show how dark-CPDs are formed in UVA irradiated melanocytes, and that FB acts as a potential antioxidant and ROS scavenger, preventing the DNA damage induced by sun exposure.

## 1. Introduction

As the largest organ of the human body, the skin, and specifically the epidermis, acts as a barrier against environmental damaging agents [1]. Among all of them, ultraviolet radiation (UVR) is one of the most extensively studied agents due to its DNA-damaging effects [1,2].

The UVR that reaches the skin can be classified into three different types according to the wavelength spectrum. UVA (320–400 nm) accounts for 95% of the UVR and has been linked to the immediate and persistent pigment darkening, as well as the generation of reactive oxygen species (ROS) [1,2]. UVB (290–320 nm) represents 5% of UVR and is associated with delayed pigment darkening and the direct alteration of DNA nucleotide structure [1,2]. While UVA and UVB rays are transmitted through the atmosphere, all UVC (100–290 nm) and some UVB rays are absorbed by the Earth’s ozone layer. During and after UVR exposure, several endogenous mechanisms are activated in order to decrease and repair the induced DNA damage [1]. Skin pigmentation is one of these photoprotective responses, which is caused by the accumulation of melanin produced by melanocytes at the epidermis [2].

Eumelanin and pheomelanin are the two predominant types of melanin produced in the skin [1,2]. Although tyrosine is the common precursor for both types, they differ in their chemical, structural and physical properties [1]. The percentage of both types of pigment is important in determining the susceptibility to DNA damage [1]. In this sense, pheomelanin is considered to be phototoxic, as it amplifies the UV-induced ROS production [2,3]. Individuals of low phototype skin in the Fitzpatrick scale, with fair skin and red hair have increased levels of pheomelanin. According to epidemiological studies, these individuals have increased risk of accumulating UVR-damage and subsequent tumor formation [1,2]. These individuals harbor inactivating polymorphisms in the melanocortin 1 receptor (MC1R), which controls pigment production. The decrease in MC1R activity is linked to a higher pheomelanin production, leading to red hair—fair skin phenotype and the associated risk of DNA damage upon UVR exposure [2]. On the contrary, eumelanin is a heterogeneous polymer that primarily acts as a photoprotectant in pigmented tissues due to the 5,6-dihydroxyindole-2-carboxylic acids (DHICAs) in its structure [2].

UVR is a complete carcinogen and has been linked to the induction of both melanoma and non-melanoma skin cancers [3,4,5]. The damaging process involves the direct absorption of both UVA and UVB photons, which triggers the dimerization of pyrimidine bases [4]. This photochemical transformation results in the formation of cyclobutane pyrimidine dimers (CPDs), the most frequent DNA alterations associated with UVR [3,4,5]. The CPDs formed during the irradiation process are known as light-CPDs [5].

An alternative mechanism of CPD formation has been described in the past few years. It consists in the formation of CPDs after UVR exposure as a result of chemiexcitation [4]. Several studies have reported that this mechanism takes place for at least 3 h after UVR exposure, with maximal CPD formation at around 2 h after irradiation [4,5,6]. These CPDs formed after light-exposure are known as dark-CPDs [4,6]. Although it happens in both melanocytes and keratinocytes, it is clear that melanin plays a key role in the generation of dark-CPDs [4,5,6,7].

**Figure 1:**
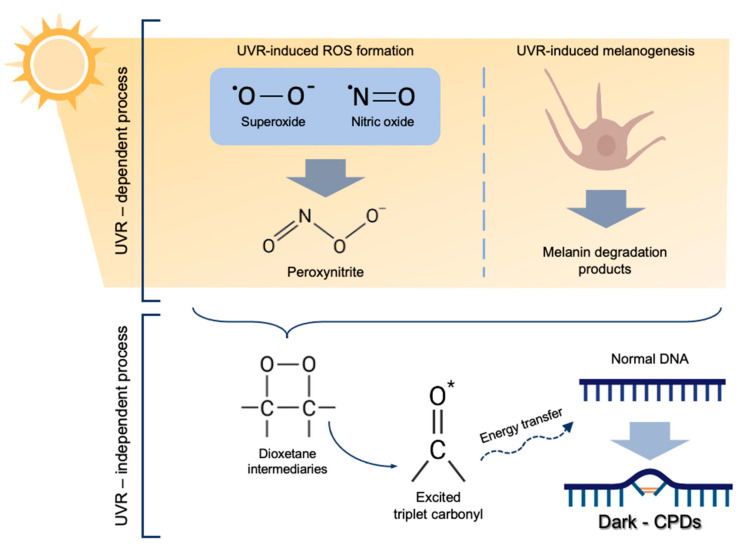
Molecular mechanism of dark-CPD formation. UVR induces ROS formation, especially superoxide and nitric oxide, which eventually form peroxynitrite. Melanin degradation products, also caused as a result of UVR react with peroxynitrite producing dioxetane intermediates, which decompose in two carbonyls, one of them being an excited-state triplet carbonyl. These excited triplet carbonyls transfer their energy to DNA bases, leading to dark-CPD formation.

The molecular mechanism underlying dark-CPD formation (Figure 1) starts with the generation of ROS and RNS, particularly superoxide and nitric oxide, as a consequence of UV exposure [6,7]. Both species undergo a chemical reaction to produce peroxynitrite, which then reacts with melanin fragments resulting from its photochemical degradation. Dioxetanes are then formed as unstable intermediaries that decompose in two carbonyls [5,6]. One of these resulting products is the excited-state triplet carbonyl, with the equivalent energy to a UV photon [6,7]. The excited triplet carbonyls transfer their energy to nearby DNA bases in a radiation-independent process, leading to the formation of dark-CPDs [5,6,7].

Dark-CPDs account for more than 50% of the total CPDs, meaning that the UVR-mediated damage to DNA occurs mostly after the light exposure [5,7]. Several studies carried out in human and mice keratinocytes and melanocytes have linked the formation of dark-CPDs specifically to UVA radiation [4,5,6,7].

Altogether, these findings point out the need of new photoprotective strategies to complement traditional sunscreens with biological filters in order to effectively protect against dark-CPD formation [5]. It has been proven that the topical use of physical and chemical filters is insufficient to avoid DNA damage caused by exposure to solar radiation [8]. In this sense, compounds capable of scavenging oxygen and nitrogen species and quenching triplet-state energy could be appropriate [7]. Hence, we propose Fernblock^®^ (*Polypodium leucotomos* extract, Cantabria Labs, Madrid, Spain) as a promising candidate to prevent the formation of dark-CPDs [8]. The composition of Fernblock^®^ could potentially reduce superoxide and nitric oxide formation, as well as the peroxynitrite [9,10]. Additionally, it increases the antioxidant cellular mechanisms, decreasing the oxidative DNA damage, and has already been reported to reduce UVB-induced CPDs [10]. In this article, we have studied the effects of Fernblock^®^ on the formation of CPDs in the dark, after the exposure to UVA light.

## 2. Materials and Methods

### 2.1. Cell Culture

The B16-F10 mouse melanocyte cell line was kindly provided by Dr. Benilde Jiménez Cuenca, Instituto de Investigaciones Biomédicas «Alberto Sols» UAM-CSIC (Madrid, Spain). Cells were cultured in Dulbecco’s modified eagle medium (DMEM) supplemented with 10% (*v*/*v*) fetal bovine serum (FBS), 1% (*v*/*v*) penicillin G (100 U/mL) and streptomycin (100 µg/mL) (HyClone Laboratories, South Logan, UT, USA). Cells were maintained under standard conditions at 37 °C, 5% humidity and 5% CO_2_ in an incubator (Heraeus HERAcell, Thermo Scientific, Waltham, MA, USA). B16-F10 cell line is a melanoma cell line and not normal melanocytes, but we consider that this cell line is appropriate for the present study. We consider that the use of a malignant cell line does not interfere with the parameters that have been evaluated in the study.

### 2.2. Cell Treatment

Fernblock^®^ (FB) is a standardized hydrophilic extract from the leaves of *Polypodium leucotomos* that has been developed to take advantage of the photoprotective properties of ferns by providing a consistent phenolic content. It was obtained as lyophilized powder from Cantabria Labs (Madrid, Spain). The extract was stored at room temperature, shielded from light, following the provider’s instructions. Stock solutions were prepared at a concentration of 10 mg/mL in distilled water, under agitation at 25–30 °C. This stock was diluted in phenol red-free DMEM 1% FBS to the desired concentrations. Previous studies published by the group have tested its efficacy against UV, visible and infrared light at concentrations ranging from 0.01 through to 10 mg/mL [10,11,12,13,14]. Based on these studies, we decided to treat cells with 0.3 and 0.75 mg/mL of FB for 24 h before UVA irradiation.

### 2.3. UVA Irradiation

Melanocytes were irradiated with UVA light with the aim of studying the effects of UVA radiation on the formation of CPDs. A CAMAG UV lamp (CAMAG, cat. no. 022.9115, El Prat de Llobregat, Spain) was used for UVA irradiation of melanocytes. Different doses of UVA irradiation were tested in order to achieve optimal CPD formation. Cells were subjected to the irradiation in 12-well plates for 1, 3, 5 and 7 min, which is equivalent to 94, 282, 470 and 658 mJ/cm^2^, respectively. Cells were irradiated in phenol red-free DMEM 1% FBS with the treatment (FB). Fresh medium was added immediately after irradiation.

### 2.4. MTT Cell Viability Assay

Cell viability was evaluated 24 h after UVA irradiation using the MTT (3-[4,5-dimethylthiazol-2-yl]-2,5-diphenyltetrazoliumbromide) assay [15]. MTT solution (100 μg/mL) was added to the cell cultures and incubated for 3 h at 37 °C. The resulting precipitate of formazan was dissolved in dimethylsulfoxide (DMSO, Panreac, Barcelona, Spain) and absorbance was measured at 542 nm using a plate reader (SpectraFluor, Tecan, Zürich, Switzerland). Data were normalized with respect to non-irradiated control values.

### 2.5. Measurement of Dark-CPD Formation

Dark-CPD formation in melanocytes was quantified by immunofluorescence 1, 2, 3, 4, 5 and 24 h after irradiation. Cells that had been grown in coverslips were fixed with formaldehyde-PBS 1X 3.7% for 30 min at 4 °C. Cells were incubated in Triton-PBS 1X 0.5% for 5 min at 4 °C in order to permeabilize the cells. Hydrolysis was performed using HCl 12.06 M for 30 min at room temperature. Samples were blocked using 20% FBS-PBS 1X for 30 min at 37 °C. Cells were incubated with the anti-CPD primary antibody (Cosmo Bio, Tokyo, Japan, Catalog No: CAC-NM-DND-001) in 5% FBS-PBS 1X (1:750) for 30 min at 37 °C. Secondary antibody incubation (AF488 Goat anti-mouse IgG, Thermo fisher, Rockford, IL, USA) was performed for 30 min at 37 °C. Samples were incubated with Hoechst33342 (Sigma-Aldrich, Darmstadt, Germany) (1:5000) for 5 min and mounted with a drop of Prolong (Invitrogen, Thermofisher Scientific, Waltham, MA, USA).

### 2.6. Measurement of NO^•^ Formation

Nitric oxide formation (NO^•^) was monitored at different time points after UVA irradiation using the Nitrite Assay Kit—Griess Reagent (Sigma-Aldrich) according to the manufacturer’s protocol. 96-well plates were used and the formation of NO^•^ was measured at 540 nm using a plate reader (SpectraFluor, Tecan, Zürich, Switzerland).

### 2.7. Measurement of O_2_^−^ Formation

Superoxide (O_2_^−^) formation was assessed at different time points using the method described by Ewing and Janeiro [16,17]. Culture supernatant was added to the reaction buffer (0.1 mM EDTA, 62 µM Nitro Blue Tetrazolium (NBT) and 98 µM NADH in 50 mM phosphate buffer pH 7.4) containing 33 µM 5-methyl phenazinium methyl sulphate in 50 mM phosphate buffer pH 7.4 containing 0.1 mM EDTA. Absorbance was measured at 560 nm using a plate reader. The NBT assay was used for this purpose, although it is not entirely specific for O_2_^−^ [18], but O_2_^−^ is the main reactive species that is quantified by this method, which gives an accurate approximation of our molecule of interest. In order to estimate how specific this method is to quantify O_2_^−^, a superoxide dismutase (SOD) inhibitable NBT reduction assay was performed by adding SOD (40U, SOD bovine, S9697-15KU, Sigma-Aldrich) to the samples for 10 min at 30 °C (see Supplementary Materials) [16].

### 2.8. Measurement of ONOO^−^ Formation

Peroxynitrite (ONOO^−^)-dependent oxidation of dihydrorhodamine 123 (DHR123) to rhodamine 123 was monitored at different time points in order to estimate ONOO^−^ production [19,20]. Cells grown in coverslips were incubated with 29 nM DHR123 for 10 min at 37 °C, shielded from light. Cells were immediately washed and visualized in a fluorescence microscope (BX-61, Olympus, Tokyo, Japan).

### 2.9. Microscopic Observations and Quantification

Microscopic observations were performed using a fluorescence microscope (BX-61, Olympus, Tokyo, Japan) with the following filter sets: blue (450–490 nm, exciting filter BP 490), green (545 nm, exciting filter BP 545). Images were obtained with an Olympus CCD DP70 digital camera. Fluorescence intensity analysis was performed using ImageJ version 1.52a (NIH, Bethesda, MD, USA). Data were normalized with respect to non-irradiated control values.

### 2.10. Statistical Analysis

Data are represented as the mean ± standard error of the mean (SEM) of at least three independent experiments. For statistical analysis, analysis of variance (ANOVA) and Bonferroni post hoc tests were run using GraphPad Prism 5.00 (GraphPad Software, Inc., San Diego, CA, USA). Differences were considered to be significant when *p* ≤ 0.05.

## 3. Results

### 3.1. Cell Viability and Selection of UVA Optimal Dose

**Figure 2:**
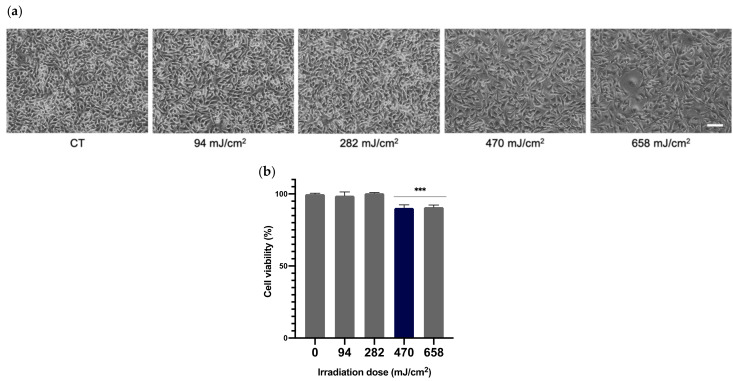
Effects of UVA radiation on the viability of melanocytes. Melanocytes were exposed to 94, 282, 470 and 658 mJ/cm^2^ of UVA radiation and micrographs were obtained 24 h after UVA radiation (**a**). Cell viability was evaluated by MTT assay performed 24 h after irradiation (*n* ≥ 3). Data were expressed as % compared to non-irradiated control cells (**b**). Data are shown as mean ± SEM. ***, *p* < 0.001. Scale bar: 50 µm.

In order to select the appropriate UVA dose that would be used throughout the whole study, viability of melanocytes after being exposed to different doses of radiation was determined. Melanocytes were irradiated at 94, 282, 479 and 658 mJ/cm^2^ of UVA and cell viability was evaluated by MTT assay 24 h after irradiation. As can be seen in the micrographs (Figure 2a) and the cell viability plot (Figure 2b), the UVA doses of 94 and 282 mJ/cm^2^ did not compromise the viability of melanocytes, compared to the non-irradiated control cells. Doses of 479 and 658 mJ/cm^2^ did, however, induce a significant decrease in cell viability (~10%).

### 3.2. Determination of Optimal UVA Dose for CPD Formation

**Figure 3:**
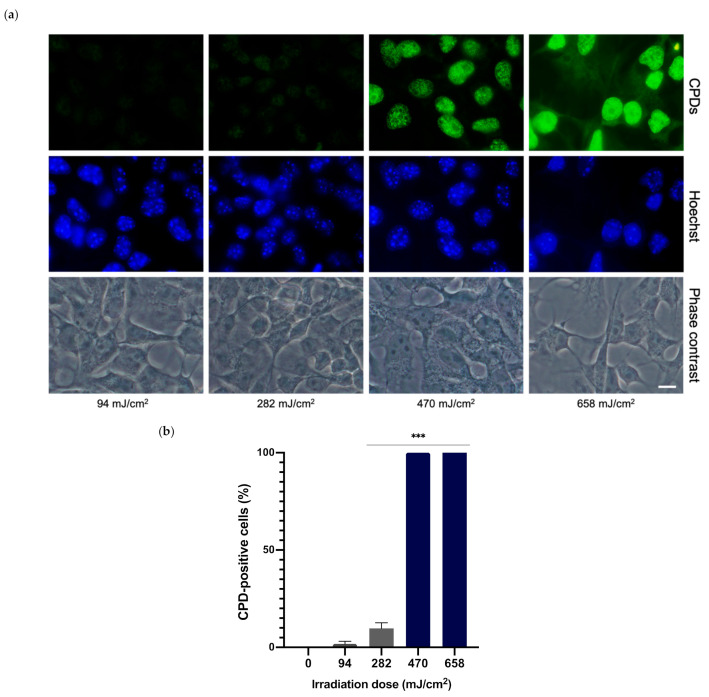
CPD formation in melanocytes exposed to UVA radiation. CPD formation was evaluated by immunofluorescence detection (**a**). Melanocytes were exposed to 94, 282, 470 and 658 mJ/cm^2^ of UVA light radiation. CPD-positive cells were counted (*n* = 5) and plotted for each dose (**b**). Data are shown as mean ± SEM. ***, *p* < 0.001. Scale bar: 10 µm.

Different UVA irradiation doses were tested in order to determine the required dose for optimal CPD formation. Melanocytes were irradiated at 94, 282, 479 and 658 mJ/cm^2^ of UVA and CPD formation was evaluated 24 h later via immunofluorescence. As can be observed in the micrographs in Figure 3a, the two lower doses tested did not induce significant CPD formation, whereas the highest evaluated dose produced high background and excessive fluorescence. The UVA dose of 470 mJ/cm^2^ induced optimal CPD formation. CPD-positive nuclei were also counted for each UVA dose (Figure 3b). The two higher doses presented 100% of CPD-positive nuclei whereas the two lower doses presented a very low percentage or no CPD-positive nuclei. Taking these results and the cell viability outcomes previously stated into account, the UVA dose of 470 mJ/cm^2^ was chosen for the remainder of the experiments.

### 3.3. Dark-CPD Formation

**Figure 4:**
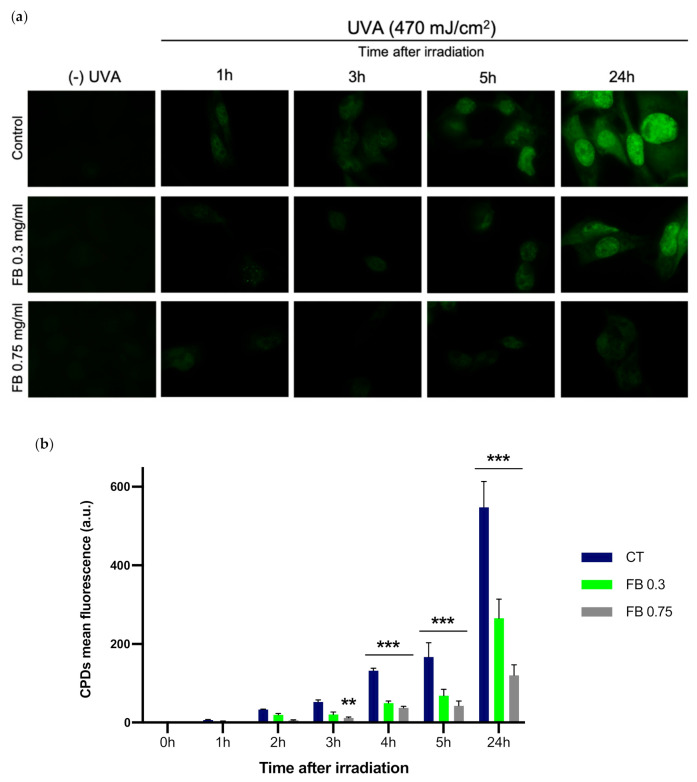
CPD formation in melanocytes exposed to UVA radiation and the effect of pre-treatment with FB. CPD formation was evaluated by immunofluorescence detection (**a**). Cells were incubated with FB 0.3 or 0.75 mg/mL for 24 h and exposed to 470 mJ/cm^2^ of UVA radiation. Quantification of the fluorescent signal from CPDs was carried out using ImageJ (*n* = 5). Data are shown as mean ± SEM (**b**). **, *p* < 0.01, ***, *p* < 0.001. Scale bar: 10 µm.

The formation of dark-CPDs in melanocytes was evaluated by immunofluorescence detection and quantified based on the mean intensity of fluorescent signal (Figure 4). Fluorescence micrographs show how UVA radiation induced the formation of CPDs, as can be observed when compared to non-irradiated controls (Figure 4a). Significant CPD formation can be detected beginning as soon as 3 h after UVA irradiation, increasing as the post-exposure time increased, reaching its highest 24 h after irradiation (Figure 4b). Pre-treatment with FB significantly reduced CPD formation, especially with the higher concentration of FB (0.75 mg/mL). Note that FB by itself did not induce any CPD formation in the control cells.

### 3.4. Formation of NO^•^

**Figure 5:**
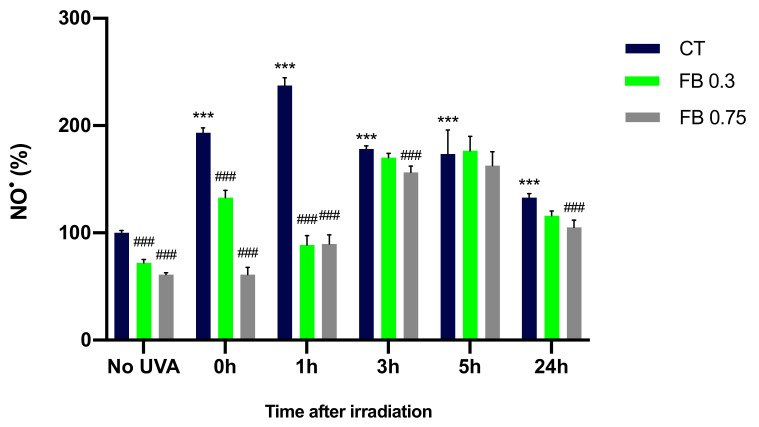
Nitric oxide formation by melanocytes exposed to UVA radiation and the effect of the pre-treatment with FB. Cells were incubated with FB 0.3 and 0.75 mg/mL for 24 h and exposed to 470 mJ/cm^2^ of UVA radiation. Nitric oxide formation was evaluated at different time points by the Griess reagent colorimetric determination (Nitrite Assay Kit—Griess Reagent, Sigma-Aldrich). Data were represented as percentages (%), taking the non-irradiated, non-treated control as reference (100%). Data are shown as mean ± SEM (*n* = 5). ***, *p* < 0.001 among CT with no FB; ###, *p* < 0.001 compared to CT among the same time point (blue bar).

In order to evaluate the production of NO^•^ after UVA irradiation and whether FB is able to prevent the increase in the production of this free radical, NO^•^ formation was evaluated in melanocytes by the Griess reagent colorimetric determination (Nitrite Assay Kit—Griess Reagent, Sigma-Aldrich) at different time points. UVA irradiation induced a significant increase in the NO^•^ formation as compared to the non-irradiated control cells (Figure 5), especially 1 h after irradiation. Levels of NO^•^ decrease after the 1 h time point, which could be explained by the fact that NO^•^ is reacting with other molecules, such as O_2_¯, decreasing the amount of NO^•^ remaining. In contrast, FB induced a significant reduction in the production of NO^•^ in melanocytes in almost all time points, especially at the concentration of 0.75 mg/mL.

### 3.5. Formation of O_2_^−^

**Figure 6:**
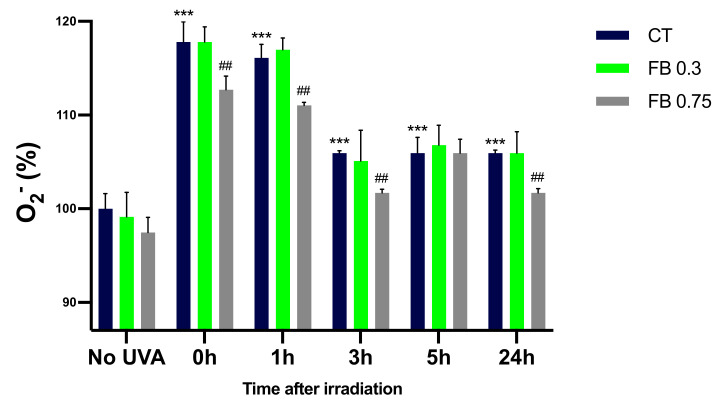
Superoxide formation in melanocytes exposed to UVA radiation and the effect of the pre-treatment with FB, evaluated using the NBT and 5-methyl phenazinium methyl sulphate method. Cells were incubated with FB 0.3 and 0.75 mg/mL for 24 h and exposed to 470 mJ/cm^2^ of UVA radiation. Data were represented as percentages (%), taking the non-irradiated, non-treated control as reference (100%). Data are shown as mean ± SEM (*n* = 5). ***, *p* < 0.001 among CT with no FB; ##, *p* < 0.01 compared to CT among the same time point (blue bar).

The NBT and 5-methyl phenazinium methyl sulphate method described by Ewing and Janeiro [16] was used in order to assess the O_2_^−^ formation in melanocytes exposed to UVA radiation. This method represents a very good approximation of O_2_^−^ quantification as seen by the SOD inhibitable NBT reduction assay that was performed (see Supplementary Materials), where very little residual NBT reduction can be seen in the samples that contained SOD, obtaining an almost complete inhibition of NBT reduction when SOD was added, meaning that O_2_^−^ is the main responsible for the NBT reduction in our experiments. As can be seen in Figure 6, irradiation of melanocytes with UVA induced a significant increase in the O_2_^−^ formation compared to control cells, especially at 0 and 1 h after irradiation. Levels of O_2_^−^ decrease after the 1 h time point, which could again be explained by O_2_^−^ reacting with other molecules. Pre-treatment with FB significantly limited the production of the superoxide anion, especially at the concentration of 0.75 mg/mL.

### 3.6. Formation of ONOO^−^

**Figure 7:**
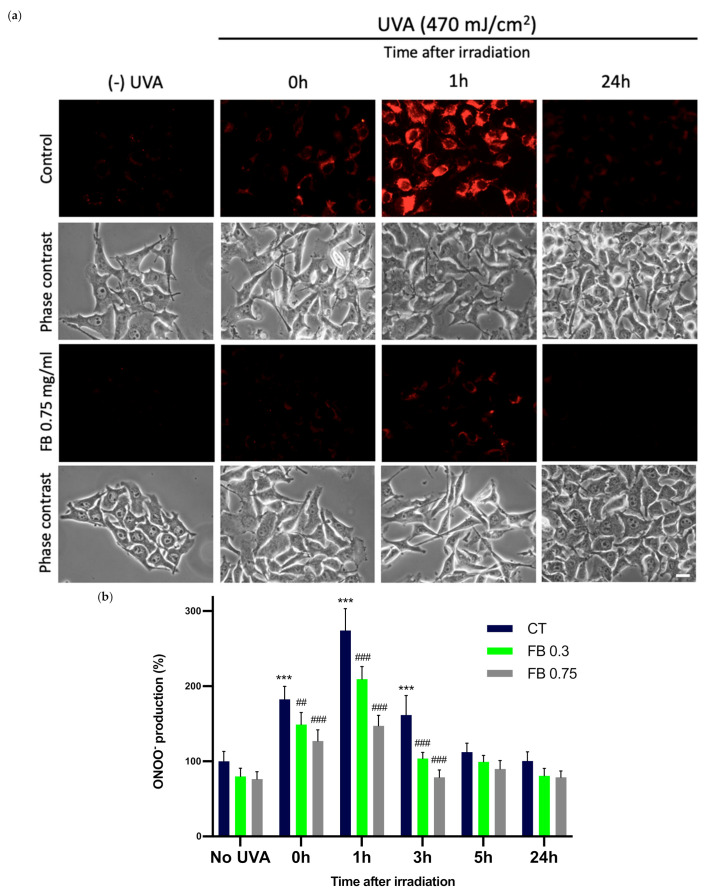
Peroxynitrite formation in melanocytes exposed to UVA radiation and the effect of the pre-treatment with FB. ONOO^−^ formation was assessed by fluorometric evaluation (**a**). Cells grown in coverslips were incubated with FB 0.3 and 0.75 mg/mL for 24 h and exposed to 470 mJ/cm^2^ of UVA radiation. Cells were incubated with 29 nM DHR123 for 10 min at 37 °C, shielded from light. Cells were immediately washed and visualized in a fluorescence microscope. Quantification of ONOO^−^ fluorescence was carried out using ImageJ (*n* = 5). Data were represented as percentages (%), taking the non-irradiated, non-treated control as reference (100%). Data are shown as mean ± SEM (**b**). ***, *p* < 0.001 among CT with no FB; ##, *p* < 0.01; ###, *p* < 0.001 compared to CT among the same time point (blue bar). Scale bar: 10 µm.

A fluorescence determination of ONOO¯ formation was performed in UVA exposed melanocytes in order to study the effect of FB on this process. ONOO^−^ formation was assessed through the evaluation of ONOO¯-dependent oxidation of DHR123 to rhodamine 123. As it can be observed in the fluorescence micrographs (Figure 7a) and by the quantification of the fluorescence in Figure 7b, UVA radiation induced an extensive magnification in ONOO^−^ formation, caused by the reaction between the increased amounts of NO^•^ and O_2_^−^ that had been formed. This increase was especially notable 1 h after irradiation of the cells, which then started decreasing. This might be related to ONOO¯ undergoing the successive reactions that eventually result in CPD formation. FB prevented the increase in ONOO^−^ production, as indicated by the reduction in fluorescence intensity, especially at the highest concentration used (shown in Figure 7a).

## 4. Discussion

The increasing amount of time people are exposed to UVR as a result of the popularization and multiplication of outdoor physical activity, recreational sunbathing and artificial tanning has given rise to serious concerns due to the many detrimental effects of this radiation [21]. UVA is responsible for the production of ROS and alterations in the DNA structure among others, increasing the risk of malignancy [1,2,21]. To help prevent this damage, pigmentation through the accumulation of eumelanin or pheomelanin, produced by melanocytes, is triggered in the epidermis [2,22,23].

UVR has been demonstrated to be responsible for the induction of skin malignancies through direct damage caused in the DNA, especially in lighter skin phototypes [19]. UVR triggers the dimerization of pyrimidine bases, leading to the formation of light-CPDs [3,4,5]. Moreover, CPD formation has also been observed to occur even after UVR exposure has ended, hence giving rise to the concept of dark-CPDs. Dark-CPDs are produced by successive reactions between ROS, RNS and melanin degradation products that lead to the formation of an excited triplet carbonyl, which transfers its energy to DNA [4,5,6,7].

Several data suggest the photoprotective effect of the standardized hydrophilic botanical extract from the fern *Polypodium leucotomos*, FB, against the detrimental effects of UV radiation on the skin. Thus, Schalka and Donato, 2019, evaluated how the addition of FB to a sunscreen formulation reduced UV-induced p53 expression in keratinocytes compared with standard sunscreens without FB, which increases as a consequence of the alteration of nuclear DNA with the generation of CPDs [8]. Also, inhibition of the accumulation of CPDs and the reduction of the sunburn cells number after FB administration were also assessed, both in clinical studies from Kholi et al., 2017 and Middelkamp-Hup et al., 2004 and some preclinical experiments [24,25,26,27]. In that regard, FB has been proposed as a potential protectant against dark-CPD formation due to its previously proven antioxidant traits [9,10]. Focusing on antioxidant activity, we recently demonstrated that FB induces detoxification enzyme activation, including several bona fide targets of Nrf2 (CAT, GPX 1 and 4, HO-1, and NQO1) promoting cellular defense mechanisms [28]. Also, previous in vitro studies proposed that UVA promotes the conversion of DHICA to IQCA in eumelanin, which then reacts with different ROS to form reactive intermediates [12,29]. Thus, the UVA-induced oxidation of DHICA-melanin is divided into two distinct but continuous stages: oxidation to IQCA and degradation to free PTCA. In this sense, we recently demonstrated that FB minimized pigment darkening preventing melanin oxidation induced by blue light [30]. Furthermore, FB has proven to have anti-tumoral effects on skin decreasing UV-induced cell proliferation and inhibiting UV-induced NF-κB and cyclooxygenase-2 (COX-2) expression, markers deregulated in skin and other epithelial cancers, among others [31]. Following the previous results from FB against CPD formation and antioxidant activity, the aim of this study was to corroborate this activity also after UVA irradiation, evaluating dark-CPD formation in time.

The results obtained in this study demonstrate that UVA induces significant dark-CPD formation as soon as 3 h after UVA exposure, although CPD formation can already be observed even 1 h after exposure. The highest peak of dark-CPD formation was obtained 24 h after exposure. These results are in concordance with previous data demonstrated by Yim et al., where highest CPD peak formation was also observed at 24 h [32]. FB significantly reduced CPD formation after exposure in a dose-dependent manner. Since dark-CPD formation can be inhibited by ROS and RNS scavengers, the effect of FB on the production of ROS and RNS was evaluated in order to decipher its action mechanism. UVA induced increased levels of production of NO^•^, O_2_^−^ and ONOO^−^ in melanocytes, which correlate with the increased levels of CPD formation. The increase in these reactive species was especially observed either immediately after or 1 h after irradiation of the cells. Pre-treatment of cells with FB reduced the elevated formation of these reactive species, proving its antioxidant and both oxygen and nitrogen species scavenging properties in a dose dependent manner. Prevention of NO^•^, O_2_¯ and ONOO¯ production by FB, might impede the formation of dioxetane intermediates that lead to excited triplet carbonyls, which are responsible for the energy transfer to DNA and the subsequent formation of dark-CPDs.

Hence, these results show how dark-CPDs are formed in UVA irradiated melanocytes through the action of successive reactions between ROS and RNS, and that FB acts as a potential antioxidant and ROS and RNS scavenger, preventing the formation of DNA damage such as CPDs, also after sun exposure. Antioxidant molecules capable of protecting after UV exposure could be added to sunscreens, since the real-use conditions of physical and chemical filters are insufficient to protect from skin damage. FB would provide a long-lasting antioxidant effect due to its skin penetration where antioxidant processes still go on even after the sunscreen is washed off the skin.

## 5. Conclusions

In summary, UVA radiation induced CPD formation in melanocytes, especially 24 h after irradiation, as seen by immunofluorescence. Pre-treatment of the cells with FB significantly reduced the dark-CPD formation to very low levels. Furthermore, UVA radiation induced an increased production of NO^•^, O_2_^−^ and ONOO^−^ in melanocytes, which can be linked to the increased formation of CPDs and dark-CPDs. FB successfully reduced the elevated production of these reactive species, proving its antioxidant and both oxygen and nitrogen species scavenging properties. Taken together, these results strongly confirm that FB is a promising candidate to complement traditional sunscreens, in order to effectively provide long-lasting skin protection against dark-CPD formation.

## Data Availability

Not applicable.

## Supplementary Materials

The following are available online at https://www.mdpi.com/article/10.3390/antiox10121961/s1, Figure S1: Superoxide dismutase (SOD) inhibitable Nitro Blue Tetrazolium (NBT) reduction assay.
