# Supplementary material for: Formation of Cyclobutane Pyrimidine Dimers after UVA Exposure (Dark-CPDs) Is Inhibited by an Hydrophilic Extract of Polypodium leucotomos
Source: Antioxidants (Basel). 2021 Dec 7;10(12):1961. doi: 10.3390/antiox10121961 (PMC8750109; doi:10.3390/antiox10121961)
Supplement: Supplementary file 1 [file antioxidants-10-01961-s001.zip › antioxidants-1482167-supplementary.pdf]

Supplementary Material

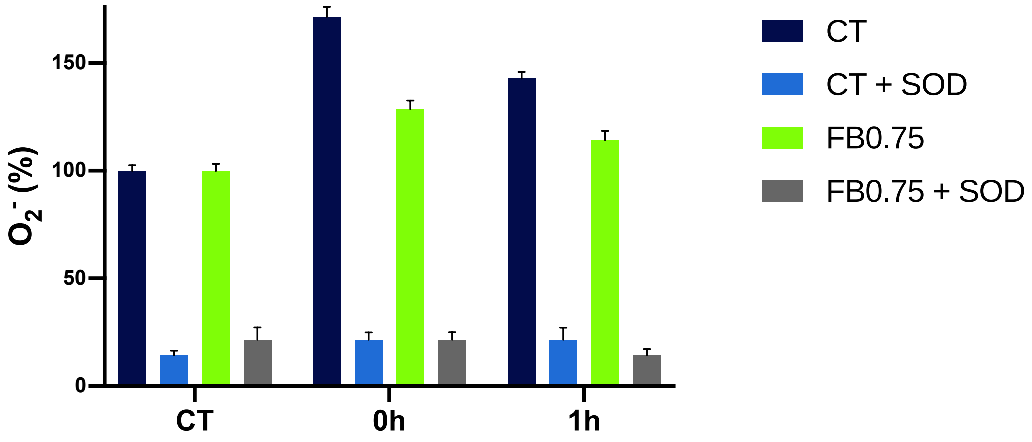

Supplementary Figure S1. Superoxide dismutase (SOD) inhibitable Nitro Blue Tetrazolium (NBT) reduction assay. Superoxide formation in melanocytes exposed to UVA radiation and the effect of the pre-treatment with FB, evaluated using the NBT and 5-methyl phenazinium methyl sulphate method. Cells were incubated with FB 0.75 mg/mL for 24 h and exposed to 470 mJ/cm2 of UVA radiation. Samples were incubated with or without SOD (40U) for 10 minutes at 30 ℃ before quantification. Data were represented as percentages (%), taking the non-irradiated, non-treated control as reference (100%).
